# Supplementary material for: Buckling mediated by mobile localized elastic excitations
Source: PNAS Nexus. 2024 Feb 16;3(4):pgae083. doi: 10.1093/pnasnexus/pgae083 (PMC10983783; doi:10.1093/pnasnexus/pgae083)
Supplement: pgae083_Supplementary_Data [file pgae083_supplementary_data.zip › PNASNEXUS-PNASNEXUS-2023-01158R-s01.pdf]

V1: High-speed videos of transient crumple dynamics for forward and return shear of sheets of narrow to wide aspect ratios. Details and timestamps are found throughout Section 3 of the main text.

V2: Normal speed video of the principal hysteresis loop studied in the paper, including stable S-ridge and O-valley pairs.

V3: Normal speed video collecting various additional phenomena mentioned in Section 3E of the main text.
